# Task- and Response Related Dissociations between Neglect in Near and Far Space: A Morphometric Case Study

**DOI:** 10.3233/BEN-2012-110243

**Published:** 2013

**Authors:** Lina Aimola, Igor Schindler, Annalena Venneri

**Affiliations:** ^1^Cognitive Neuroscience Research Unit, University of Durham, DurhamUK; ^2^Clinical Neuroscience Centre, University of Hull, HullUK; ^3^Department of Neuroscience, University of Sheffield and Sheffield Teaching Hospital, Sheffield, UK and San Camillo Hospital, VeniceItaly

**Keywords:** Spatial neglect, distance, line bisection, visual search, MRI, VBM

## Abstract

Introduction: Patients with unilateral neglect may show line bisection errors selectively in either near (within hand reaching) or far (beyond hand reaching) space which suggests that these two spatial areas are coded differently by the brain. This exploratory study investigated, whether any difference in performance between these spatial domains might be task-independent or modulated by the requirement for a motor response.

Methods: A 31-year-old right brain damaged patient (MF) and a group of age matched healthy controls were assessed with two serial visual search tasks and a Landmark paradigm. Both types of task required either a directional (pointing) or non-directional (button press) motor response. Participants were assessed with both task types and response modes in near (57 cm) and far space (114 cm).

Results: MF showed left neglect during visual search only in far space for the perceptual condition and in near space for the motor condition. MF showed no neglect in both versions of the Landmark task irrespective of spatial distance. A voxel-based morphometric assessment of MF's brain lesion showed marked damage in the right ventro-temporal cortex, superior temporal gyrus, insula, inferior frontal gyrus, angular gyrus and bilaterally in the posterior cingulate cortex.

Conclusions: Our preliminary findings suggest that processing of far space during visual search is associated with ventral stream damage but only when space is coded through visual information. Neglect involving directional motor activity in near space seems to be associated with damage of structures sharing close connections with the dorsal stream.

## 1. Introduction

Visuospatial neglect is a debilitating condition that usually follows right hemisphere damage. The patients affected by this syndrome fail to report, respond, explore or orient toward stimuli predominantly located in the contralesional hemispace in the absence of any primary sensory or motor deficits [1]. The severity of the deficit may not only vary depending on the type of task used but also on whether stimuli are presented within (near) or beyond (far) reaching space. Visuospatial neglect in relation to space distance has been assessed primarily with tests of line bisection. The results, however, are far from consistent. Several studies have found a marked decrease in bisection error from near to far space [2–4]. Others have found the opposite pattern with larger bisection errors in far space than in near space [5–9]. Within a group of 28 unselected right brain damaged patients, some individual cases of neglect were found, that were restricted to either near or far space [10]. However, these effects of distance were not significant when the data were analysed at the group level.

ISSN 0953-4180/13/$27.50 © 2013 – IOS Press and the authors. All rights reserved

Comparatively very few studies have used visual search tasks to investigate neglect in near and far space. In a single case study, a decrease of marked left neglect from far to near space was observed [11]. In the two group studies that have been carried out to investigate the influence of distance in neglect, no systematic effect of this factor has been found on performance during visual search. In one instance, a space by position interaction, with greater impairments in far space than in near space, was reported [12], whereas no influence of distance on visual search performance in a cancellation task was found by the other study [9].

Taken together, the findings of these studies appear to suggest that line bisection tasks are more sensitive to uncover distance related neglect, whereas neglect related visual search deficits seem to be less modulated by spatial distance [9]. Dissociations between neglect in near and far space, with either task, have been repeatedly reported when performance of individual cases has been looked at.

Furthermore, the cognitive operations involved by these two tests are different and it is not uncommon that patients might show neglect in one task but not in the other [13–16]. Thus, it remains to be clarified whether neglect for near and far space is a task-independent phenomenon or if in the same patient the type of task used can modulate the magnitude of neglect within near or far space.

Another potentially confounding aspect in modulating neglect severity across distance concerns the type of response required, i.e. whether a task is performed purely on a perceptual basis or whether it requires a motor response. Some tasks typically require a directional motor response (e.g. manual line bisection tasks) while others only require verbal feedback of a perceptual judgment of the stimuli (e.g. Landmark paradigms, using pre-bisected line stimuli).

For example, a study which used the Wundt-Jastrow area illusion test to assess perceptual neglect found no dissociation between neglect in near and far space [17]. In contrast, dissociations between near and far space were reported in studies that used manual line bisection tasks. It was then suggested that near and far space representation may be based on output-related mechanisms [3,4,6–8,11,17]. In other words, the representation of near and far space may be dependent on the preparation and execution of specific motor acts implemented upon those space sectors which, in turn, could influence the processing of sensory stimuli [18,19].

More recently, it has been suggested that distance-based deficits in neglect patients may occur even in the absence of a directional manual response [12,20]. Using manual and verbal versions of the line bisection task, even in the absence of significant group effects, a distance effect on neglect was found, regardless of response type, when the performance of individual patients was examined [20]. When a verbal visual search task was used, more severe deficits in far than in near space were detected, even when patients were not required to perform a directional manual response [12]. These findings suggest that the presence of a motor act when performing a task may not be necessary to detect distance-related behavioural dissociations.

Overall, this inconsistency in findings is partially due to the relatively small number of studies which did not use a directional motor response toward the stimuli. Most importantly, except for the study reviewed above [20], to our knowledge there are no within-subject studies in which performance in near and far space has been assessed using the same test, with and without a directional motor response.

Here we report the case of patient MF who, following right hemisphere damage, showed a varying pattern of neglect in near and far space. In the present study, we evaluated whether the severity of neglect in near and far space is task dependent (line bisection vs. visual search) and whether it is altered by the presence/absence of a directional motor response.

## 2. Case report

MF is a right-handed man (31-year old at time of testing) with 20 years of formal education, who works as a manager in a local bar. He has no background history of alcohol or substance abuse. In 2004, at the age of 28, he became the victim of a violent street attack during which he fell down and hit the side of his head on a kerbstone after being punched in the face. As a consequence, MF suffered traumatic brain injury with damage to the right ventro-medial and superior temporal areas, insula, inferior frontal gyrus, angular gyrus and bilateral posterior cingulate cortex (see results section for details). MF was unconscious at admission to hospital (3/15; Glasgow Coma Scale) and remained in a mild coma for two weeks out of a total of eight weeks of hospitalisation. Although his skull was not fractured, his acute CT scan showed haemorrhagic contusions of the right temporal lobe (substantial) and right subfrontal cortex (minimal) and required surgery for removal of the subdural haematoma.

Earlier medical reports stated that after his accident MF developed post-traumatic amnesia which lasted about two weeks, some twitching of the left upper and lower limbs, and of the left side of his face, mild constructional apraxia and substantial concentration difficulties. No visual field deficits were present as assessed by dynamic perimetry. MF never experienced any episodes of epilepsy nor did he show any signs of seizure in a series of 24 hour EEG recordings (last carried out in 2006).

His family noticed a mild change in his personality after the accident and described him as more irritable, inclined to lose his temper easily and absentminded during his daily activities.

In 2007, MF was referred for a comprehensive neuropsychological follow-up examination. MF scored 33/36 on the Raven's Progressive Matrices and showed no impaired performance on Verbal Paired Associates Learning (14/24), Digit Span (8) and the Token task (33/35). His scores on Category (number of words: 55) and Letter Fluency (number of words: 35) tasks were within the normal age range. He showed mild impairment in his performance on the Rey-Osterreith Complex Figure, and omissions of some of the items were present both in direct copying (30/36) and in delayed recall (9.5/36). Omissions, however, were not lateralised. MF scored normally on the Visuoconstructive Apraxia test (13/14). There was a selective attention deficit detected on the Digit cancellation task, with 7 non-lateralized omissions out of 60 targets.

During interview, when questioned about his daily life, MF casually reported that his customers often complained about him serving and taking orders more readily from some but not others. Further detailed questioning revealed that he would more readily attend to customers standing to his right, whereas he would serve those standing to his left only if verbally prompted. He was not particularly troubled by these problems and he spontaneously commented: 'I don't know what they have to complain about, they all get served eventually if they shout!'. He also reported in response to specific questions that during his everyday activities he would frequently run into doors or pieces of furniture. He was, however, unconcerned about these difficulties, and despite his relatives and friends' expressing concern for these problems, MF appeared largely unaware of his difficulties. MF's self-reported ecological observations about his workplace prompted the experimenters to further investigate the possibility that some of his everyday difficulties might be due to spatial neglect.

### *2.1. Assessment of neglect*

The battery used to assess spatial neglect included the following five clinical standard tests: the Bells test [21], Star Cancellation and line bisection from the Behavioural Inattention Test [22], Scene Copying [23] and representational drawing (clock face). Given the difficulties reported by the patient, the presence of neglect was also assessed in far space. For this purpose, only the cancellation and line bisection tests were administered with an overhead projector positioned behind the patient, 140 cm away from the wall and with the stimulus size controlled for visual angle. In this case MF gave his responses by using a laser pointer.

Overall, there was mild neglect, and deficits were more prominent in far space than in near space, especially for cancellation tasks. In detail, in near space MF showed no impairment in line bisection (error $= +0.04^\circ$) and no omissions in representational drawing or Scene Copying (5/5). There were three target omissions in the Bells test (32/35) but none in the Star Cancellation task (54/54). In far space MF's rightward bisection error increased slightly to $+0.2^\circ$ and there was an increased number of omissions in the cancellation tasks (Bells test: 28/35; Star Cancellation task: 51/54).

## 3. Methods

### *3.1. Control sample*

In all experimental tasks, MF's performance was compared with that of 10 healthy, right-handed controls (NC) matched for age, gender and education (Mean age = 30.5 years, SD = 2.11; Mean education = 21.4 years, SD = 2.71). All healthy participants gave their informed, written consent to take part in the study and had no history of psychiatric disorders, brain damage, epilepsy or drug addiction. None was taking any medications at time of testing.

### *3.2. Tasks*

#### *3.2.1. Landmark line bisection*

This line bisection paradigm was adapted from Milner, Brechmann and Pagliarini [24]. Participants were presented with white, horizontal lines ($20^\circ \times 1^\circ$ of visual angle) in the centre of a black screen. In each trial, the line was transected vertically at one of nine Landmark positions (0, 5, 15, 30, 60 mm to the left or the right of the objective line centre). Participants were asked to make a judgment about the length of the two line segments. In one session they were asked to judge which end of the line was shorter and in another session they had to decide which end was longer. The two kinds of judgment ('shorter' or 'longer') were requested for the same stimuli in two separate blocks of trials, and served to control for a possible response bias. Six stimuli per Landmark transection were presented, 54 trials in total per block (shorter or longer). One hundred and eight trials were presented overall in each distance condition (near or far).

Distance conditions (near and far) and type of judgment (shorter and longer) were counterbalanced across participants. All stimuli were presented randomly, one at a time, and stayed on screen until a response was given. No central fixation point was presented between trials and, following each response, the next stimulus appeared after a 1 s delay.

*3.2.2. Balloon search task*

The visual search task was a modified computerised version of the Balloon test version B [25]. The target was a single circle (2° × 1° of visual angle) among 35 distractors consisting of circles of the same diameter with an adjoining vertical line. Each stimulus was presented in white against a black background. Eighteen stimuli were presented within each visual field and could appear randomly across trials in one of 36 locations within a 6 × 6 virtual array. Participants were asked to respond with their right hand when they detected a target. Each trial, was preceded by a fixation condition in which four dots (0.5° × 0.5°) converged over 400 ms from the corners of a virtual square (4° × 4°) to a single fixation dot (0.5° × 0.5°) at the centre of the display. This was intended to draw attention to the centre of the array in preparation for the upcoming trial. The inter-trial interval was 500 ms. There were 180 trials overall, with 45 trials (including 9 catch trials) for each distance condition (near/far) and response mode (motor/perceptual).

*3.3. Design and procedure*

All the tasks were administered in two distance conditions (near and far). The distance between the participant's head and the stimuli projected was 57 cm for the near space condition and 114 cm for the far space condition. Stimuli were presented via a Sony Vaio laptop (17" monitor) connected to an Epson LCD projector located behind the participant. The visual angle was kept constant during the two distance conditions (60.31° × 40.21°). All tasks were carried out in complete darkness to minimize the influence of any visual cues in the surrounding environment. The centre of the display was aligned with the participant's sagittal plane. Participants carried out two versions of each task which will be referred as 'perceptual condition' and 'motor condition'.

In the perceptual version of both experimental tasks participants were asked to respond by pressing two different buttons on a keyboard for stimuli presented in near and far space. In the Balloon task they had to press the number '1' key for the 'target present' response and the '0' key for the 'target absent' response. In the Landmark task, participants were required to press the number '1' key to indicate the left segment and the number '2' key for the right segment (for both 'shorter' or 'longer' responses). In the motor conditions participants were instructed to make a movement with their right arm toward the target (visual search task) or the chosen segment (Landmark task) by touching them with their right index finger in near space and by pointing at them with a laser pen in far space. In the visual search task, if they thought that the target was absent they were asked to verbally inform the experimenter who recorded the responses on the computer. The Landmark task required always a forced-choice judgement even for the centrally bisected lines.

Type of task, response type and distance were counterbalanced and each experimental condition was preceded by 10 practice trials. Participants were instructed to respond as accurately and as quickly as possible.

*3.4. Follow-up testing*

MF's stability of performance was re-examined 8 months after the first testing session and compared with that of the same group of controls as in his initial assessment. The basic experimental set-up and Landmark task were identical to the first testing session. His visual search abilities were assessed by using a different search paradigm (see below) which is more akin to classical cancellation tasks than the Balloon task.

*3.4.1. Letter search task*

Participants were presented with a search display of white items [0.5° × 0.5° of visual angle] against a black background. The display consisted of a random array of 10 single-digit target letters together with 96 distractors (78 symbols and 18 numbers) which were distributed randomly for each trial. Thus, for each presentation 106 stimuli were shown with five targets in each hemifield. A total of 120 trials were administered with 30 trials in each combination of response modality (perceptual and motor) and spatial condition (near/far). The inter-trial interval was set at 500 ms and contained a central fixation point ($0.5° \times 0.5°$) to encourage central fixation at the onset of the next trial. In the perceptual condition, participants were asked to name all the letters they could find. For the motor condition, they were instructed to touch the targets with their right index finger in near space and point at the letters with a laser pen in far space. Participants were instructed to explore the display carefully and press the space bar on the computer keyboard to trigger the presentation of the next trial when they thought they had found all the targets. For each trial, the number of correctly identified targets was recorded by the experimenter together with the total exploration time as defined by the participants' keyboard responses.

### *3.5. Behavioural data analysis*

Data from MF were compared with those of controls using computerised versions of the modified t-tests proposed for the study of single cases [26]. The Sokal and Rolph modified t-test [27] was used to verify whether MF's score achieved in each spatial distance was significantly lower than that of the controls. The Revised Standardized Difference Test [28] was applied for comparing the difference in MF's performance between the sets of scores in near and far space with the distribution of differences observed in the control group. The analysis of the Landmark task was carried out using the method of Toraldo and co-workers [29] who proposed two mathematically independent indices disentangling 'perceptual' and 'response' biases. The point of subjective equality (PSE, here transformed in degrees of visual angle) between two sections of a bisected line represents the perceptual bias. The response bias index (M), on the other hand, represents the probability that a response will be made in the opposite direction of the subject's PSE (i.e. the tendency to favour 'right' responses, regardless of the perceptual experience of the stimulus). A negative PSE score indicates a left perceptual bias, whereas a positive value implies a right perceptual bias. The same principle applies to the M index: a positive score signifies a right response bias and vice versa.

## 4. Results

### *4.1. First session*

#### *4.1.1. Landmark task*

MF's response bias was not significantly different from the controls' M-index in near and far space in neither the perceptual nor the motor condition ($t(9) \leq 1.92$, $p > 0.05$). In addition, his response bias did not change significantly across distances in both the perceptual ($t(9) = 0.00$, $p > 0.05$) and the motor ($t(9) = 0.58$, $p > 0.05$) conditions [MF: −0.07–0.09, NC Mean: −0.02–(−)0.01].

When asked to judge which end of the line was shorter (or longer), MF's showed a leftward perceptual bias which did not differ from that of controls for either distance [near: $t(9) = -1.24$, $p > 0.05$; far: $t(9) = -1.15$, $p > 0.05$]. Similarly, when asked to point at one end of the line, no significant difference between the patient's leftward PSE score and that of controls was found in both distance conditions [near: $t(9) = -0.14$, $p > 0.05$; far: $t(9) = -0.70$, $p > 0.05$]. MF's PSE amplitude did not change significantly across space distances compared with the control group in both the perceptual ($t(9) = 0.07$, $p > 0.05$) and the motor version ($t(9) = 0.76$, $p > 0.05$) of the task [MF: −2.50°–(−)0.63°; NC Mean: −1.01°–(−)0.38°].

#### *4.1.2. Balloon search task*

The accuracy data and response times were analysed separately for the perceptual and motor condition in three steps. In a first step each subject's overall percentage correct responses and response times were collapsed across the left and right visual fields and MF's data compared with those of the control group in near and far space. In a second step, to verify whether MF showed an asymmetrical search pattern, MF's difference values (percentage correct/response times in the right visual field minus those in the left visual field) between the visual fields were compared with the mean difference values of the control group for each space distance. In a final step, these difference values were used to compare the change in MF's accuracy/response time bias from near to far space with that of the control group.

Table 1
Balloon task: Mean percentage (SD) of correctly detected targets and response times in seconds for MF and normal controls (NC) as a function of task condition (perceptual, motor), distance (near, far) and visual field (left, right)

| | Accuracy perceptual | | | | Accuracy motor | | | |
|---|---|---|---|---|---|---|---|---|
| | Near | | Far | | Near | | Far | |
| | L | R | L | R | L | R | L | R |
| MF | 67.00 | 67.00 | 67.00 | 78.00 | 83.00 | 100.00 | 89.00 | 89.00 |
| NC | 96.17 (4.37) | 96.17 (4.37) | 98.00 (2.95) | 97.17 (4.45) | 98.08 (3.68) | 97.58 (3.80) | 97.50 (3.09) | 98.08 (3.68) |
| | Response times perceptual | | | | Response times motor | | | |
| MF | 3.85 | 3.96 | 3.05 | 4.08 | 5.17 | 4.69 | 5.07 | 5.34 |
| NC | 3.29 (0.53) | 3.60 (0.63) | 3.33 (0.60) | 3.53 (0.80) | 3.45 (0.36) | 4.41 (0.82) | 3.92 (0.84) | 4.14 (1.21) |

L: Left; R: Right.

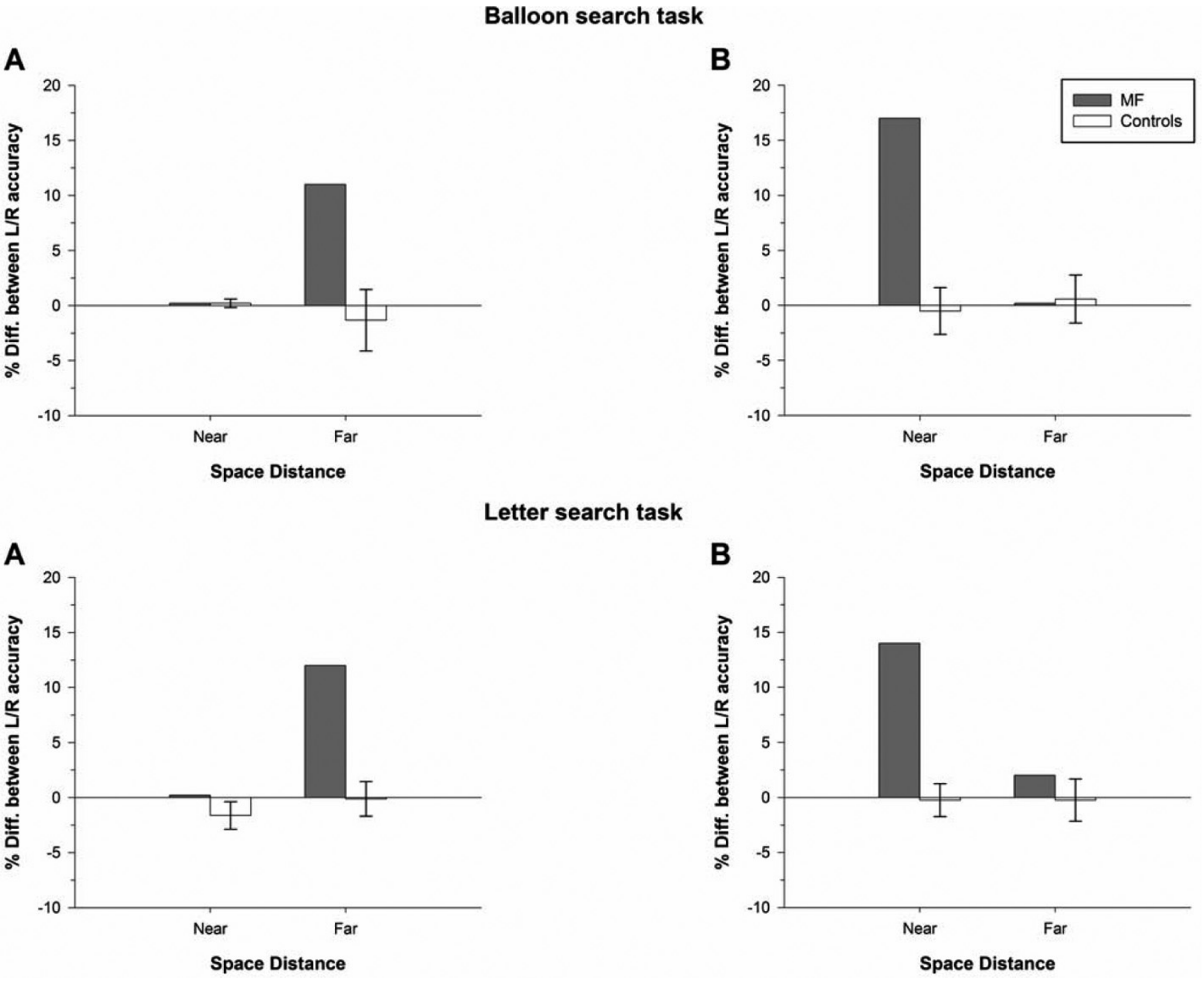

Fig. 1. Difference in accuracy (+/− 2 SEM) between the left and right visual field in near and far space for MF and the controls. Positive/negative values indicate a rightward/leftward bias in accuracy in the (A) perceptual and (B) motor condition.

*Perceptual condition*

Overall MF detected fewer targets in near space than did the controls [MF = 67%; NC = 96%; $t(9) = -7{,}06$, $p < 0.001$], but showed no left/right bias in comparison with the controls ($t(9) = 0.0$, $p > 0.05$), detecting the same number of targets in each visual field (see Table 1 and Fig. 1). In far space, MF still detected fewer targets than the controls did [MF = 72%, NC = 97.6%; $t(9) = -7{,}19$, $p < 0.001$], but there was a rightward bias in his performance, detecting 11%

more targets on the right, while the controls showed a small leftward bias ($-2\%$) ($t(9) = 3.54$, $p < 0.01$). MF's difference in patterns of performance between hemifields across near and far space was significantly different from that of controls ($t(9) = 2.37$, $p < 0.05$).

MF's response times were comparable with those of the controls in both near and far space and no specific left/right bias was found (see Table 1).

*Motor condition*

As in the perceptual condition MF's overall accuracy was significantly lower than that of controls in both near ($t(9) = -1.88$, $p < 0.05$) and far space ($t(9) = -3.41$, $p < 0.01$) (Near: MF = 92%; NC = 98%; Far: MF = 89%, NC = 98%). When the right/left hemifield difference was taken into account, in near space, MF's accuracy was significantly biased toward the right visual field (17%) compared with controls who showed a small leftward bias in detecting 2% more targets on the left ($t(9) = 4.48$, $p < 0.01$). In contrast, in far space MF was equally accurate in both hemifields and there was no significant difference from controls ($t(9) = -0.12$, $p > 0.05$) (see Table 1). This decrease in bias from near to far space was significantly different from that shown by controls whose difference in accuracy between the two visual hemifields remained consistent in near and far space ($t(9) = 4.34$, $p < 0.001$).

As with the perceptual condition MF's overall response times were not significantly different from controls in both near and far space [Near: MF = 3.08 sec, NC = 3.13sec; Far: MF = 3.50 sec, NC = 3.20 sec; $t(9) \leq 1.75$, $p > 0.05$]. In near space, however, MF showed a rightward bias and was significantly slower in detecting stimuli in the left than in the right hemifield ($t(9) = -2.16$, $p < 0.05$). In far space, there was no difference between the two hemifields ($t(9) = 0.05$, $p > 0.05$). The difference in MF's response times in favour of the right visual field decreased significantly from near to far space compared with controls whose performance remained stable ($t(9) = 1.80$, $p < 0.05$).

## 4.2. Follow-up session

The data analysis followed the same methods used for analysis of the tasks in the first testing session. However, for the Letter search task, only the data of 8 controls were used as two participants did not complete all the experimental conditions.

### 4.2.1. Landmark task

MF's pattern of performance was very similar to that of the first testing session: neither in the perceptual nor in the motor condition did MF show a significant motor bias in either near or far space when compared with controls [MF = $-0.06$–0.04, NC Mean = $-0.01$–0.03; $t(9) \leq 1.25$, $p > 0.05$]. In addition, there was no evidence that MF's slight leftward perceptual bias was different from the PSE of the controls across response modalities or spatial distance [MF = $-2.06°$–$(-)1.56°$, NC Mean = $-1.25°$–$(-)0.50°$; $t(9) \leq 1.43$, $p > 0.05$].

MF's normal pattern of performance as assessed by the M-index and the PSE parameters in the Landmark task remained stable across spatial distances and response modalities ($t(9) \leq 2.14$, $p > 0.05$).

### 4.2.2. Letter search task

MF's exploration times in the two space distances were not significantly slower than those of the control group, neither in the perceptual ($t(7) = 0.39$, $p > 0.05$) nor in the motor condition ($t(7) = 0.788$, $p > 0.05$) (see also Table 2).

*Perceptual condition*

Overall MF's accuracy was significantly lower than that of the controls in near space [MF = 88%; NC = 95%; $t(7) = -3.18$, $p < 0.05$], but he showed no bias in target detection towards the left or right visual fields when compared with controls ($t(7) = 0.87$, $p > 0.05$).

In far space, MF not only detected fewer targets than the controls [MF = 82%; NC = 96%; $t(7) = -7.27$, $p < 0.0001$] but he also showed a clear rightward search bias detecting 12% more targets in the left hemifield than in the right hemifield, whereas the controls showed a balanced search performance ($-0.12\%$) ($t(7) = 0.51$, $p < 0.005$). This change in hemifield bias from near to far space was significantly different from controls ($t(7) = -3.00$, $p < 0.05$).

*Motor condition*

MF's overall accuracy in the motor condition was not different from that of the controls in near space [MF 91%, NC 95%; $t(7) = -1.56$, $p > 0.05$] but was significantly lower in far space [MF 87%; NC: 95%; $t(7) = -5.802$, $p < 0.005$]. However, in near space, MF showed a significant rightward bias (14% more targets on the left), performing nearly at ceiling in the right visual field whereas controls were equally accurate in both visual fields ($-0.22\%$; see also Fig. 1). In contrast, he only detected 2% more targets in the

Table 2
Letter search task: Mean percentage (SD) of correctly detected targets for MF and normal controls (NC) as a function of task condition (perceptual, motor), distance (near, far) and visual field (left, right). The mean search times in seconds (SD) are shown for each task condition and distance

| | Accuracy perceptual | | | | Accuracy motor | | | |
|---|---|---|---|---|---|---|---|---|
| | Near | | Far | | Near | | Far | |
| | L | R | L | R | L | R | L | R |
| MF | 88.00 | 88.00 | 76.00 | 88.00 | 84.00 | 98.00 | 86.00 | 88.00 |
| NC | 96.38 (2.07) | 94.75 (2.71) | 96.13 (1.96) | 96.00 (1.69) | 95.25 (3.01) | 95.00 (2.39) | 95.13 (1.55) | 94.88 (2.17) |
| | Search times perceptual | | | | Search times motor | | | |
| MF | 20.64 | | 24.66 | | 24.63 | | 34.44 | |
| NC | 23.99 (12.73) | | 22.37 (75.28) | | 37.47 (41.44) | | 24.27 (10.07) | |

L: Left; R: Right.

Table 3
Areas of grey matter volume loss in patient MF compared with healthy controls

| Brain area | Right/Left | Brodmann area | Cluster size | Talairach coordinates X | Y | Z | Z-value at local maximum |
|---|---|---|---|---|---|---|---|
| Inferior temporal gyrus | R | 20 | 56 | 44 | −7 | −16 | 2.61 |
| Insula | R | 13 | 194 | 44 | −36 | 22 | 2.51 |
| Angular gyrus | R | 39 | | 42 | −51 | 23 | 2.42 |
| Superior temporal gyrus | R | 41 | | 38 | −30 | 14 | 2.18 |
| Cerebellum | L | | | −18 | −62 | −31 | 2.46 |
| Posterior cingulate | R | 31 | 169 | 12 | −45 | 30 | 2.40 |
| | R | 23 | | 8 | −38 | 26 | 2.27 |
| Inferior frontal gyrus | R | 9 | 56 | 50 | −1 | 24 | 2.33 |
| Precentral gyrus | R | 6 | | 48 | −16 | 25 | 2.17 |
| Fusiform gyrus | R | 19 | 30 | 42 | −47 | 4 | 2.19 |
| | R | | 16 | 20 | −38 | −27 | 2.14 |
| Cerebellum | R | | | 26 | −46 | −31 | 2.11 |

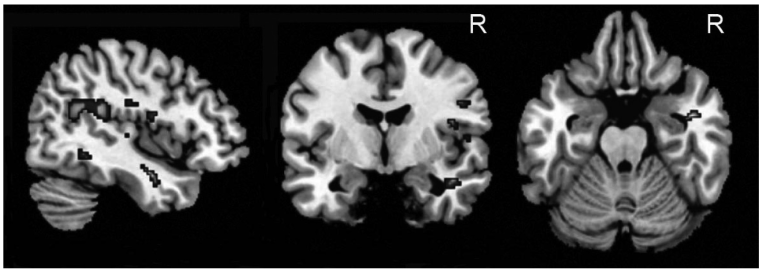

Fig. 2. Areas of grey matter volume loss in patient MF relative to controls. Differences are superimposed on a high resolution T1W structural MRI scan. Slices are positioned at $x = 43$ (sagittal), $y = -6$ (coronal) and $z = -18$ (axial).

right compared with the left hemifield in far space, and this performance was not significantly different from that of controls (−0.22%) ($t(7) = 0.82, p > 0.2$). This change from a rightward- to no bias across the two spatial distances was significant compared with the unchanged performance in the control group ($t(7) = -3.97, p < 0.05$).

### 4.3. Brain morphometry: MRI acquisition, pre-processing, statistical analysis and results

Three dimensional T1-weighted MRI images were acquired on a 3.0 T GE MRI system with a SPGR sequence using a standard head coil. Voxel dimensions were 0.547 × 0.547 × 1.50 mm. The field of view was 240 mm with a matrix size of 512 × 512 × 140. A number of pre-processing steps were followed to isolate grey and white matter from the 3D T1-weighted structural scans before performing statistical analyses using SPM5 (The Wellcome Trust Centre for Neuroimaging, UCL, London, UK).

To correct for global differences in brain shape, structural images were warped to standard stereotactic space and segmented to extract grey matter, white matter and cerebrospinal fluid. The grey and white matter segments were then modulated to correct for

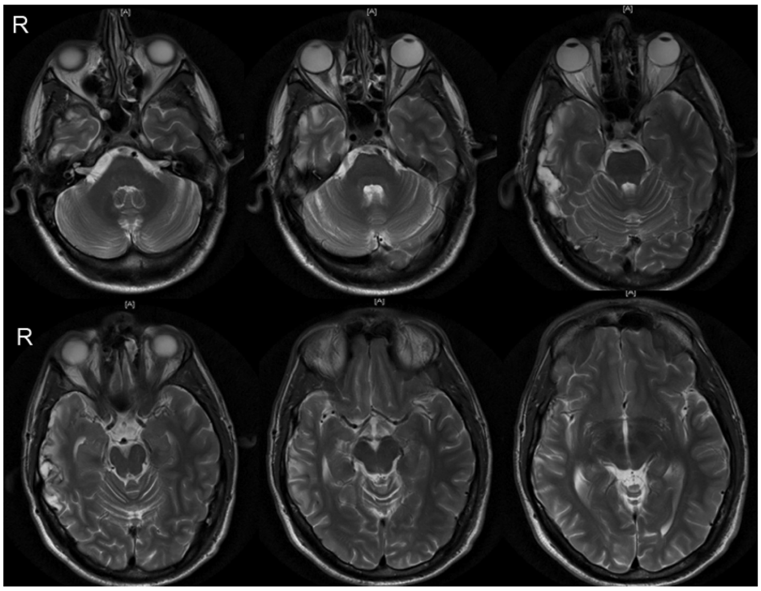

Fig. 3. MF's T2W scan showing damage in right temporal cortex. The image is shown in radiological orientation (R/L).

changes in volume induced by nonlinear normalisation and smoothed using a Gaussian filter set at 8 mm to reduce possible error from between-subject variability in local anatomy and render the data more normally distributed. Smoothed grey and white matter segments were entered into the analyses. Anatomical regions were identified using the Talairach Daemon Client (http://www. talairach.org/), following conversion of the Montreal Neurological Institute coordinates extracted from the SPM analyses into Talairach coordinates using the Matlab function mni2tal (http://imaging.mrc-cbu.cam.ac.uk/downloads/MNI2tal/mni2tal.m). Height threshold was set at $p < 0.05$ (uncorrected) with an extent threshold of at least 5 voxels. Only voxels surviving FWE corrections were considered significant.

Voxel-based independent t-test comparisons between MF's grey and white matter segments and those of 9 controls (5 males, 4 females) matched for age (Mean = 34.44 years, SD = 11.52) and education (Mean = 19.22 years, SD = 3.03) were carried out. Areas of grey matter volume loss were found in the right ventro-temporal and superior temporal cortex, insula, inferior frontal gyrus, angular gyrus and posterior cingulate cortex (see Fig. 2 and Table 3 for details). There were no areas of significant white matter volume differences between MF and the controls.

Axial T2 weighted scans were also acquired. The pattern of tissue damage shown by these images broadly paralleled the more detailed findings of the morphometric analyses (see Fig. 3).

## 5. Discussion

Patient MF showed impairments that were restricted to the effective exploration and detection of relevant targets during serial visual search. In contrast, his capacity to judge the horizontal extent of two segments of a single line stimulus remained intact. His deficit was more severe in far space when the task involved a perceptual response, but the opposite was true in near space when target detection had to be indicated via a motor response.

The dissociation between neglect in the Landmark task and the visual search tasks is in line with the findings of studies that have compared performance on classic line bisection tasks with cancellation tasks. Several studies have shown that performance on these two types of task dissociates and correlates poorly. It has also been suggested that these tasks not only engage different cognitive processes, but they might also be subserved by different brain structures [30–33]. Line bisection or perceptual size judgment tasks seem to involve mainly posterior (occipito-parietal) brain regions [30,34,35] which were spared in MF. In contrast, visual exploration deficits appear to be associated with damage in a more anterior neural network, including right temporal, insular and frontal cortex [30]. This neural network was partially damaged in MF.

Although MF showed relatively mild neglect there was a clear differential effect of distance and response requirement on his search bias. This modulatory effect of response type on the severity of neglect for near and far space has been observed in other studies that have investigated the effect of tool use on performance on line bisection (e.g. [2]). Neglect confined to near space extended also to far space when the patient was asked to perform the task with a stick rather than with a laser pointer. The use of a tool appeared to trigger some remapping of what was the intact far space as impaired near space [2,36]. This could explain why some studies found no difference in performance between near and far space in visual search tasks [9]. In the current study, even without the use of a tool, MF's space-related dissociation in visual search changed only by introducing or removing a directional motor response of his upper limb.

This behavioural pattern can be interpreted within different theoretical frameworks. In proximal space, the operations of grasping and visuomotor coordination have greater importance than in distal space where the ability to visually recognise, explore and discriminate relevant features in the environment appears more relevant as it is not supported by touch [37]. This idea is compatible with neurophysiological findings in monkeys showing that peripersonal space is represented mostly by bimodal neurons with visuotactile receptive fields [38–40]. Extrapersonal space representation, on the other hand, relies primarily on neurons with visual receptive fields [18,41]. It has been suggested that near space attentional mechanisms recruit spatial reference frames based mainly on limb/hand movements while far space attention might depend more on oculomotion in the upper visual hemispace [42,43]. It follows that a task requiring not only visual scanning of the stimuli but also a directional motor response towards the stimuli (such as the motor version of the Balloon task) would be more likely to reveal impairment within near space. When the same task requires visual exploration without a directional motor response (such as the perceptual version of the Balloon task), deficits in far space would be more detectable. The pattern observed in patient MF fits with this suggestion. MF had an impaired representation of both sectors of space but the severity of this impairment did become manifest only when assessed with specific testing procedures. It appears, therefore, that a visuospatial impairment for a specific sector of space can be elicited by motor actions engaging different effectors (i.e. limb or eye movements) or a combination of both, through which attention is allocated in space. This interpretation is compatible with the premotor theory of attention which states that the coordinate frames in which space is coded in the brain depend on the motor requirement and the effectors [18, 19]. Accordingly, spatial attention would rely on sensorimotor neuronal networks involved in the transformation of spatial information into a specific action goal [44].

Alternatively, it is conceivable to consider MF's asymmetrical performance in far space as prevalently due to a perceptual impairment that improved in the motor condition with the recruitment of additional reference frames based on the hand and upper limb. MF's pointing movements could also have acted as an attentional visuomotor cue which allowed him to overcome his perceptual visuospatial deficit. The idea that a conscious representation of space can be modulated through a combination of visual and motor cueing of attention to the affected side has been proposed by Forti and Humphreys [45]. These authors reported a patient who showed less severe neglect during a visual search task when a stick was used, but also when he searched with his ipsilesional arm outstretched or with a torch. The authors concluded that motor actions combined with a visual cue (the arm) may enhance attention and detection at the location for which an action is programmed. Within this framework, in the motor condition MF's performance in far space may have improved through the effect of a visuomotor cue represented by the full view of his arm and the action of pointing with a laser pen. In near space the patient may have benefited less from this cueing effect as in this condition he could only see his hand and part of his forearm during the reaching action. Although possible, the conditions of darkness in which the patient was tested weaken the plausibility of such an interpretation.

The patient's pattern of performance could also be interpreted by speculating on what mechanisms may have been disrupted based on the location of his brain damage. MF's lesion involved the right ventrotemporal and superior temporal cortex, insula, inferior frontal gyrus, angular gyrus and the posterior cingulate cortex bilaterally. Damage involving the ventrotemporal and fusiform structures may be related to MF's ipsilesional search bias in far space. This hypothesis is supported by another single case of neglect for visual search tasks which was more severe in far space following damage of the right medial and inferior temporal regions [11]. The brain areas damaged in both cases represent major components of the ventral stream which is assumed to be critically involved in the representation of extrapersonal space [46,47]. The ventral stream is specialised for colour processing and complex feature integration in far space and these types of fine local analyses are less important during reaching operations in near space that are mediated primarily by the dorsal visual stream [46,47].

MF's impairment in near space was present only when he had to make a ballistic motor response towards targets in the contralesional hemifield during visual search. The nature of MF's exploration deficit involving reaching movements in near space might be related to damage of brain structures that are closely connected with the dorsal stream, such as the angular gyrus and the posterior cingulate cortex. There is evidence that the angular gyrus is a core brain region that when damaged causes neglect in near space [31]. This area seems to participate in the voluntary orienting of visual attention and corporeal awareness by maintaining a stable body representation [48,49]. Most importantly, this structure is involved in coding space within an egocentric frame of reference for exploratory goal-directed motor behaviour that relies on near space representations [48–50].

The posterior cingulate cortex is also active during reflexive saccades [51] and covert attention processes involving anticipatory shifts of visual attention [52].

Interestingly, anatomical studies in humans and monkeys found strong connections between the posterior parietal cortex and the dorsal part of the posterior cingulate cortex suggesting a relay function of the posterior cingulate cortex within the dorsal stream (for details see [53]). These findings are supported by evidence from neuroimaging studies that have reported activation in this region associated with visual feedback during reaching, pointing and grasping [54, 55]. Based on these findings it is, therefore, conceivable that damage of the posterior cingulate cortex in MF may have selectively affected his visual exploration abilities while carrying out contralateral reaching movements within near space.

MF showed also damage of the right superior temporal- and insular cortex that have been found to be damaged in lesion group studies that have investigated neglect using cancellation tasks [32]. The superior temporal cortex is located at the transition between the two visual streams and has been suggested to represent an important cortical site where information from both pathways is integrated [56]. Oram and Perrett [57] demonstrated, that in monkeys the cells of the superior temporal cortex code for both objects properties (i.e. shape) and their direction of motion. Thus, the superior temporal cortex may play an important role in the reconstruction and updating of the surrounding space as a coherent whole by integrating object-related features with their position relative to the viewer. The insular cortex is also thought to be critical for integrating vestibular and neck proprioceptive inputs to build multimodal spatial representations that are impaired in neglect [32]. It is possible, therefore, that both the superior temporal- and insular cortex are involved in the representation of near and far space during visual search.

As for the inferior frontal cortex, some studies have found that neglect patients with frontal damage perform poorly on high density cancellation tasks with a relatively preserved performance in line bisection tasks [30,58,59]. This pattern can be explained by the critical role played by the right inferior frontal lobe in target selection and information filtering (i.e. irrelevant distractors in a search task). Accordingly, damage to the frontal cortex may have contributed to MF's poor performance in the visual search task in near and far space and the absence of any impairment in the Landmark task. In addition, similarly to the superior temporal cortex, the prefrontal cortex may be another critical brain site where the integration of information carried by both high visual pathways may take place [60]. There are indeed cells in the prefrontal cortex that code both the identity of visual stimuli (ventral stream inputs) and their spatial location (dorsal stream inputs) [61,62]. Within the near space/dorsal stream and far space/ventral stream framework [63], the converging inputs from both streams in the superior temporal and prefrontal cortex (both damaged in MF, although to different extents) may explain the dissociation of MF's visual exploration deficit in both space domains depending on response mode.

Taken together, the available anatomical evidence in MF shows that damage of the ventral stream results in far space neglect only when space is coded through perceptual information (i.e. visual), while the occurrence of neglect when acting within near space might be associated with the angular gyrus and posterior cingulate cortex damage in close connection with dorsal stream structures.

In conclusion, our preliminary findings suggest that there is a complex interaction between individual lesion patterns, task and mode of response underlying dissociations in neglect for near and far space, and that this complex interaction can modulate behaviour even in a case that on standard assessment showed a pattern of typical neglect. It would be interesting to apply the methods used in this single case study to a larger group of patients with typical neglect to clarify more fully how this complex interaction modulates the expression of neglect symptoms.

## Acknowledgments

The authors thank MF for the time devoted to this study and the MRI centre personnel at Hull Royal Infirmary who helped with the 3D MRI scanning.
